# *Eimeria ovinoidalis* Infection Reshapes Gut Microbial Communities and Metabolic Profiles in Tan Sheep

**DOI:** 10.3390/biology14091190

**Published:** 2025-09-04

**Authors:** Jiandong Wang, Yuxi Zhao, Pan Wang, Youli Yu, Yarong Xu, Yuqiu Yang

**Affiliations:** 1 Institute of Animal Science, Ningxia Academy of Agricultural and Forestry Sciences, Yinchuan 750002, Chinayyl06010323@163.com (Y.Y.); 2 School of Animal Science and Technology, Ningxia University, Yinchuan 750021, Chinaxyr_0305@163.com (Y.X.); yangyuqiu0729@163.com (Y.Y.)

**Keywords:** Tan sheep, *Eimeria ovinoidalis*, coccidiosis, gut microbiome, metagenomics, metabolomics, microbial diversity, host–pathogen interaction

## Abstract

Tan sheep, a valuable indigenous breed in China, are vulnerable to coccidiosis caused by *Eimeria ovinoidalis*. In this case-control study, four 8-month-old Tan sheep raised under identical conditions were enrolled, including two with confirmed *E. ovinoidalis* infection (the Eo group) and two healthy controls (the HC group). Integrated metagenomic and untargeted metabolomic analyses were performed to assess gut microbiota and metabolic alterations. Results showed reduced alpha diversity and a distinct microbial composition in the Eo group. LEfSe identified 38 differentially abundant bacterial species, with *Prevotella* sp. and *Fusobacterium necrophorum* enriched in the Eo group and *Faecalibacterium* sp. and *Lachnospira* sp. enriched in the HC group. KEGG and VFDB analyses revealed significant differences in microbial functional pathways and virulence factor profiles. A total of 543 metabolites were differentially expressed, involving pathways related to inflammation, stress response, and amino acid metabolism. Microbiome–metabolome correlation analysis showed that Eo-associated bacteria were positively linked to pro-inflammatory metabolites, while HC-associated taxa correlated with markers of metabolic homeostasis. These findings provide new insights into the pathogenesis of ovine coccidiosis and may inform targeted interventions.

## Simple Summary

Tan sheep are an important native breed in China that frequently suffer from a parasitic disease called coccidiosis, which damages their intestines and reduces their health and productivity. Understanding how this disease affects the gut bacteria and chemical processes in infected animals could help develop better treatments and prevention strategies. In this study, the gut microbiome and metabolic profiles of infected and healthy Tan sheep were compared to identify the biological changes caused by the parasite. The results show that infected sheep had different bacterial communities in their guts, with more harmful bacteria and fewer beneficial ones, compared to healthy animals. The infected sheep also showed altered levels of various metabolic compounds, particularly those involved in inflammation and stress responses. These bacterial and chemical changes were closely connected, suggesting that the parasite disrupts the normal balance of gut microbes, which then affects the sheep’s metabolism and immune responses. These findings enhance our understanding of how coccidiosis develops and progresses, and may help researchers and veterinarians design more effective treatments that both target the parasite and restore a healthy gut function in affected animals.

## 1. Introduction

Ovine coccidiosis, caused by *Eimeria* species, represents a major parasitic disease affecting global sheep production, with resulting economic losses exceeding billions annually [1]. Infection poses particular challenges for Tan sheep (*Ovis aries*), a premium indigenous breed renowned for exceptional fur quality and environmental adaptability that serve as a cornerstone of livestock agriculture in China’s Ningxia Hui Autonomous Region. The infection primarily targets young animals, manifesting as profuse watery diarrhea, dehydration, and compromised growth performance [2,3]. In severe cases, infections progress to hemorrhagic enteritis with mortality rates reaching 20–30% in untreated flocks [3]. Sheep are susceptible to several *Eimeria* species, including *E. crandallis*, *E. ovina*, *E. faurei*, *E. parva*, and *E. granulosa*, among others [4,5]. Among the pathogenic species, *Eimeria ovinoidalis* (*E. ovinoidalis*) is the most virulent, predominantly colonizing epithelial cells in the ileum, cecum, and colon [6,7]. Unlike *Giardia* (associated with chronic, greasy diarrhea with bloating), *E. ovinoidalis* causes acute hemorrhagic enteritis with severe dehydration [7,8].

The gut microbiota—a complex microbial community encompassing bacteria, archaea, fungi, and viruses [9]—plays a crucial role in immune system development and disease susceptibility. Through interactions with intestinal lymphoid tissueNC, microbiota regulate immune cell maturation and function [10,11] while producing metabolites, such as short-chain fatty acids (SCFAs), that maintain intestinal homeostasis [12]. Coccidial infections substantially disrupt this delicate ecosystem by altering key metabolic pathways including unsaturated fatty acid biosynthesis, teichoic acid biosynthesis, and butanoate metabolism, as demonstrated in Hu lambs infected with *E. ovinoidalis* [7]. During infection, antimicrobial metabolites like lividamine are upregulated while anti-inflammatory compounds such as butyric acid are significantly reduced, potentially exacerbating inflammation and compromising gut barrier integrity [7].

These local microecological changes trigger systemic effects through the gut–systemic axis [7,13]. Compromised intestinal barriers allow endotoxins such as lipopolysaccharides to enter the bloodstream, inducing systemic inflammation characterized by elevated pro-inflammatory factors (IL-6, TNF-α) and decreased anti-inflammatory mediators (IL-10) [13]. This systemic response can affect distant organs, including the reproductive system, highlighting coccidiosis as a disease with both local and systemic consequences [7,13].

Despite growing recognition of the impact of coccidial infection on gut microbiota, most studies remain limited to single-omics analyses, which hinders comprehensive understanding of host–microbiome interactions. High-throughput sequencing technologies and multi-omics approaches offer unprecedented opportunities to elucidate complex molecular mechanisms underlying parasitic infections [14,15,16,17]. The integration of metagenomics and metabolomics enables the construction of comprehensive microbial databases and the identification of specific microbe–metabolite associations that may serve as biomarkers or therapeutic targets [18,19,20]. However, such integrated approaches have rarely been applied in veterinary parasitology due to technical complexity, cost constraints, and data integration challenges.

To address these limitations, we performed combined metagenomic and metabolomic analysis of fecal samples from coccidia-infected and healthy Tan sheep. By integrating information about microbial community composition, functional profiles, and metabolite signatures, our objectives were to (1) characterize the gut microbiome and metabolic disturbances induced by coccidial infection, (2) identify key microbial taxa and metabolites associated with infection severity, and (3) investigate potential microbe–metabolite interactions driving pathogenesis. These findings advance our fundamental understanding of host–pathogen–microbiota dynamics and support the development of microbiota-informed diagnostic and therapeutic strategies for ovine coccidiosis.

## 2. Materials and Methods

### 2.1. Study Design

This case-control study was conducted in Ningxia Hui Autonomous Region, China, in January 2025. Four 8-month-old Tan sheep maintained under identical feeding conditions were enrolled—two animals naturally infected with *Eimeria ovinoidalis* (Eo group) and two healthy controls (HC group). Fresh fecal samples were collected from all animals and subsequently subjected to parasitological and molecular analyses, as described in Section 2.2 and Section 2.3.

### 2.2. Parasitological Methods

Fecal oocyst counts were estimated using the saturated saline flotation technique combined with a McMaster counting chamber. Approximately 2 g of fecal material were homogenized in 28 mL of saturated sodium chloride solution (specific gravity 1.18–1.20) and thoroughly mixed. A small aliquot of the suspension was loaded into a McMaster chamber, and Eimeria oocysts were counted under a light microscope at 100× magnification. Oocyst counts were expressed as oocysts per gram of feces (OPG) by multiplying the observed number by the dilution factor according to standard procedures [21]. The average oocyst load in the infected group was approximately 5 × 10^6^ oocysts per sample.

### 2.3. Molecular Methods

Genomic DNA was extracted from approximately 200 mg of each fecal sample using the DNA Stool Kit (Tiangen Biotech, Beijing, China) according to the manufacturer’s protocol. Total RNA was isolated from 200 mg of fecal material using the Stool RNA Kit (Omega Bio-Tec, Norcross, GA, USA), and complementary DNA (cDNA) was synthesized using the PrimeScript RT reagent Kit (Takara, Shiga, Japan) using random hexamer primers.

PCR screening was performed to exclude *Cryptosporidium* spp. [22], *Giardia duodenalis* [23], and *Rotavirus* [24]. For coccidia species identification, an internal transcribed spacer (ITS)-targeted PCR was used [25]. The primer sequences, annealing temperatures, and amplification conditions are provided in Supplementary Table S1.

### 2.4. Microbiome Sequencing and Bioinformatics Analysis

In this case-control study, genomic DNA was isolated from fecal samples collected from the Eo group (*n* = 2) and the HC group (*n* = 2). For each fecal sample, DNA extraction and sequencing were performed in triplicate to ensure technical reproducibility, resulting in a total of 12 sequencing datasets. These triplicates represent technical replicates of the same biological samples rather than independent biological replicates. DNA extraction was performed using the OMEGA Mag-Bind Soil DNA Kit (M5635-02, Omega Bio-Tek, Norcross, GA, USA) following the manufacturer’s protocol, with DNA integrity assessed via agarose gel electrophoresis and concentration and purity quantified using a Qubit 4 Fluorometer (Qubit protein assay kit, Invitrogen, Waltham, MA, USA). Metagenomic libraries were prepared according to the Illumina TruSeq DNA Sample Preparation Guide, targeting insert sizes of approximately 400 bp, and paired-end sequencing (2 × 150 bp) was performed on the Illumina HiSeq 4000 platform (Illumina, San Diego, CA, USA).

Raw sequencing reads underwent quality control using Trimmomatic v0.36 (The Usadel Lab, Aachen University, Aachen, Germany) [26,27] followed by host genome removal using Bowtie2 v2.2.9 (Johns Hopkins University, Baltimore, MD, USA) [28] alignment against the sheep reference genome. Quality-filtered reads were assembled into contigs using MEGAHIT v1.2.9 (https://github.com/voutcn/megahit) (accessed on 15 February 2025) [29], and open reading frames (ORFs) were predicted with Prodigal v2.6.3 (https://github.com/hyattpd/Prodigal) (accessed on 15 February 2025) [30]. A non-redundant gene catalog was constructed using CD-HIT v4.6.7 (https://github.com/weizhongli/cdhit/releases/tag/V4.6.7) (accessed on 15 February 2025) [31] with 95% identity and 90% coverage thresholds, retaining the longest representative sequence per cluster, and gene abundance was quantified by mapping cleaned reads to the gene catalog using Bowtie2 v2.2.9 (http://bowtie-bio.sourceforge.net/bowtie2/index.shtml) (accessed on 15 February 2025) with a 95% identity threshold.

### 2.5. Taxonomic and Functional Analysis

Taxonomic classification was performed against the NCBI non-redundant (nr) database (https://ftp.ncbi.nlm.nih.gov/blast/db/FASTA/) (accessed on 15 February 2025), and metagenomic sequences were subjected to taxonomic classification using DIAMOND v.0.9.30 (Max Planck Institute for Biology Tübingen, Tübingen, Germany; University of Dundee, Dundee, UK) [32] against the NCBI non-redundant genes with functional databases [33], generating abundance profiles from the domain to species level based on gene expression.

Alpha diversity metrics and abundance profiles were calculated and visualized using R v4.3.2 (R Core Team, Vienna, Austria), with statistical comparisons between groups employing the Wilcoxon rank-sum test (*p* < 0.05) and differentially abundant taxa identified using Linear Discriminant Analysis (LDA) Effect Size (LEfSe) with significance LDA score thresholds of >2 and *p* < 0.05 [34].

Functional profiling was conducted through DIAMOND alignment to the Kyoto Encyclopedia of Genes and Genomes (KEGG) database v20230830 (https://www.genome.jp/kegg) (accessed on 15 February 2025) with an E-value threshold of 1 × 10^−5^ [35]. Moreover, potential virulence determinants were assessed via alignment with the expertly assembled catalog of bacterial pathogenicity genes available in the Virulence Factor Database (VFDB) v20240301 (http://www.mgc.ac.cn/VFs/main.htm) (accessed on 15 February 2025) [36].

### 2.6. Untargeted Metabolomic Analysis

A global metabolomics approach was employed following established protocols [37]. Fecal specimens underwent metabolite extraction via methanol-mediated protein removal followed by chromatographic separation using a Vanquish UHPLC platform (Thermo Fisher Scientific, San Jose, CA, USA) coupled with a UPLC BEH Amide analytical column was purchased from Waters (Milford, MA, USA). Data acquisition files were transformed into mzXML format through ProteoWizard software v3.0.8789 (ProteoWizard Software Foundation; Palo Alto, CA, USA) and subsequently subjected to computational processing using the *xcms* package (v3.4.2) (Bioconductor Project, La Jolla, CA, USA) [38] in the R environment for feature detection, chromatographic alignment, and signal integration. Compound annotation was achieved through comparison against a proprietary tandem mass spectrometry reference library. Orthogonal partial least squares discriminant analysis (OPLS-DA) was applied to assess metabolomic profile differences between the experimental groups. Differentially expressed metabolites were identified based on the following criteria: variable importance in projection (*VIP*) > 1, *p*-value < 0.05, and |*log*_2_*FC*| > 1. Biological pathway mapping and functional enrichment were executed using the KEGG repository v20230830 (https://www.genome.jp/kegg) (accessed on 15 February 2025) to interpret the metabolic perturbations observed between the experimental groups.

### 2.7. Statistical Analysis 

Pairwise comparisons were performed using unpaired t-tests or Wilcoxon rank-sum tests, while multiple group comparisons were performed using one-way ANOVA or Kruskal–Wallis tests, depending on data distribution. Correlations between datasets were assessed using Spearman’s rank correlation coefficients. All analyses were conducted in R v4.3.2 (R Core Team, Vienna, Austria), with two-tailed *p*-values and significance thresholds of * *p* < 0.05, ** *p* < 0.01, and *** *p* < 0.001.

## 3. Result

### 3.1. Metagenome Profiling

Metagenome sequencing generated a total of 1,161,349,114 reads, with 96,779,092.83 ± 2,338,418.33 reads (mean ± standard error of the mean [SEM]) per sample (Table S2). After quality control and removing host genes, a total of 636,851,456 reads were retained, with 53,070,955 ± 6,747,681 per sample (Table S3). After de novo assembly, a total of 4,734,141 contigs were generated (the N50 length of 1937 ± 535 bp), with 394,512 ± 92,886 per sample (Table S4). The metagenome consisted of 96.34% bacteria (317,835,330 sequences), 1.89% archaea (6,221,584 sequences), and 1.70% viruses (5,594,792 sequences), 0.06% eukaryotes (39,390 sequences) (Table S5). Given that bacteria accounted for an overwhelming majority of the sequencing data, subsequent analyses primarily focused on the structural and functional characteristics of the bacterial community.

### 3.2. Alpha and Beta Diversity Analysis

To assess variations in microbial community diversity across clinical states, we conducted comprehensive alpha and beta diversity analyses.

**Figure 1:**
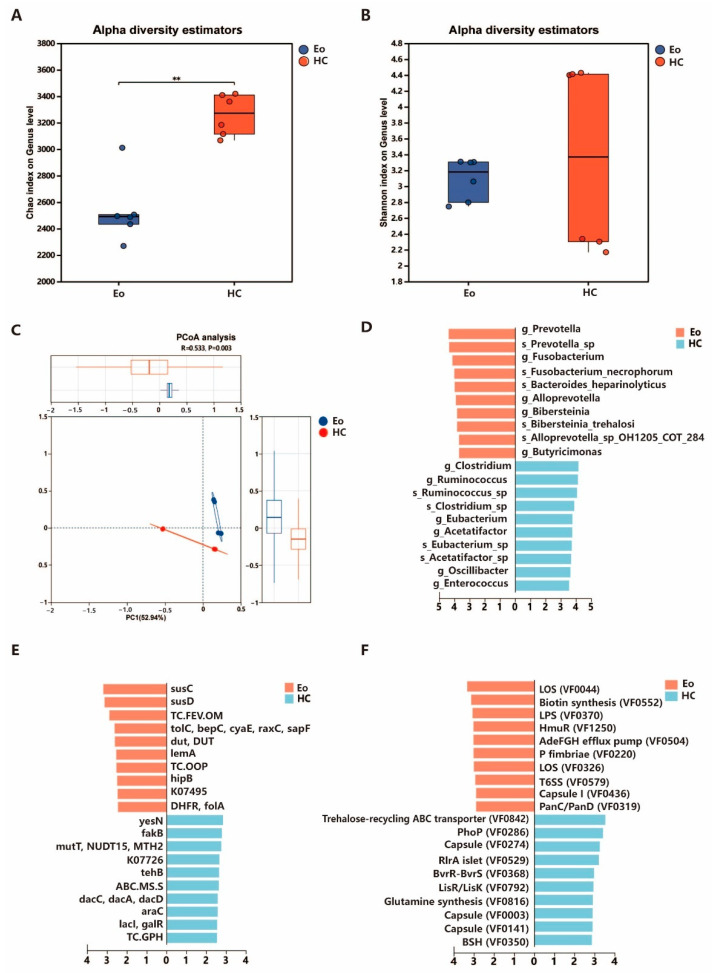
Gut microbiota diversity and functional differences across two groups. (**A**,**B**) Alpha diversity measured by Chao and Shannon index at the genus level. (**C**) Beta diversity Principal Co-ordinates Analysis (PCoA) at the genus level comparing Eo and HC groups. (**D**) LEfSe analysis identifying the top 10 differentially abundant bacteria taxa between Eo and HC groups. (**E**) KEGG pathway enrichment analysis showing the top 10 different functions between Eo and HC groups. (**F**) Top 10 different virulence factors between *Eimeria ovinoidalis* (Eo group) and healthy controls (HC group) groups.

For the overall composition of the gut microbiome at the genus level, we observed that the alpha diversity (Chao index) was significantly lower in the Eo group compared to the HC group (Wilcoxon *p* < 0.01) (Figure 1A); however, no significant difference was found in the Shannon index between the two groups (Wilcoxon *p* > 0.05) (Figure 1B). Principal coordinate analysis (PCoA) based on the Bray–Curtis dissimilarity of microbial composition showed significant difference between the structure of the gut microbiome of the two groups (Figure 1C).

### 3.3. Abundance and Composition of Different Bacteria Between Groups

To identify specific bacterial taxa associated with the Eo and HC groups, we performed a taxonomic comparison of gut microbiota between using LEfSe. The LEfSe analysis identified 45 bacterial taxa that were significantly enriched between the Eo and HC groups, with LDA effect size scores ranging from 3.015 to 4.376 (LDA > 3, *p* < 0.05), indicating distinct microbial community signatures characteristic of each group. The Eo group was predominantly enriched with ten genera, most notably *Prevotella* (LDA score = 4.376, mean abundance = 4.739) and *Fusobacterium* (LDA score = 4.130, mean abundance = 4.411) along with multiple *Alloprevotella* species, *Bacteroides heparinolyticus* (LDA = 3.981), *Neisseria* (LDA = 3.643), *Mediterraneibacter*, *Bibersteinia*, *Butyricimonas*, *Porphyromonas*, and *Streptococcus*. In contrast, the HC group demonstrated significant enrichment in 16 bacterial genera, predominantly butyrate-producing taxa including *Clostridium* (LDA = 4.168, mean abundance = 4.578), *Ruminococcus* (LDA = 4.135, mean abundance = 4.456), and *Eubacterium* (LDA = 3.771) as well as other beneficial commensal bacteria such as *Blautia*, *Oscillibacter*, *Ruminiclostridium*, *Butyrivibrio*, *Faecalibacterium*, *Enterococcus*, *Acetatifactor*, and *Agathobacter* (Figure 1D) (Table S6).

### 3.4. Functions Different Between the Eo and HC Sheep

The functions of the microbiome were determined using the KEGG profiles and genes encoding VFDB.

When the identified KEGG functions were compared, the LEfSe analysis revealed a total of 195 significantly different functional pathways between the Eo and HC groups (LDA score > 2, *p* < 0.05). Among these, 93 KEGG functions were significantly enriched in the Eo group, including susC, susD, and TC.FEV.OM. In contrast, 102 KEGG functions were predominantly abundant in the HC group, including cbh, spoIIP, vanSB/ vanS/vanSD), and cwlO (Figure 1E) (Table S7).

A comparison of the virulence factor profiles based on the VFDB annotation revealed distinct patterns between the Eo and HC groups. The LEfSe analysis with the criteria of LDA > 2.0 and *p* < 0.05 identified 104 virulence factors that were significantly different between the groups. Specifically, 48 virulence factors were significantly enriched in the HC group, including capsule-associated loci and regulatory systems such as PhoP, LisR/LisK, and BvrR-BvrS, as well as trehalose-metabolism- and glutamine-synthesis-related elements. Similarly, 56 virulence factors were significantly enriched in the Eo group, with higher LDA scores observed for lipooligosaccharide (LOS), lipopolysaccharide (LPS), biotin synthesis, and adherence-related components such as P fimbriae and Type VI secretion system (T6SS) (Figure 1F) (Table S8).

### 3.5. Non-Target Metabolomics Analysis

**Figure 2:**
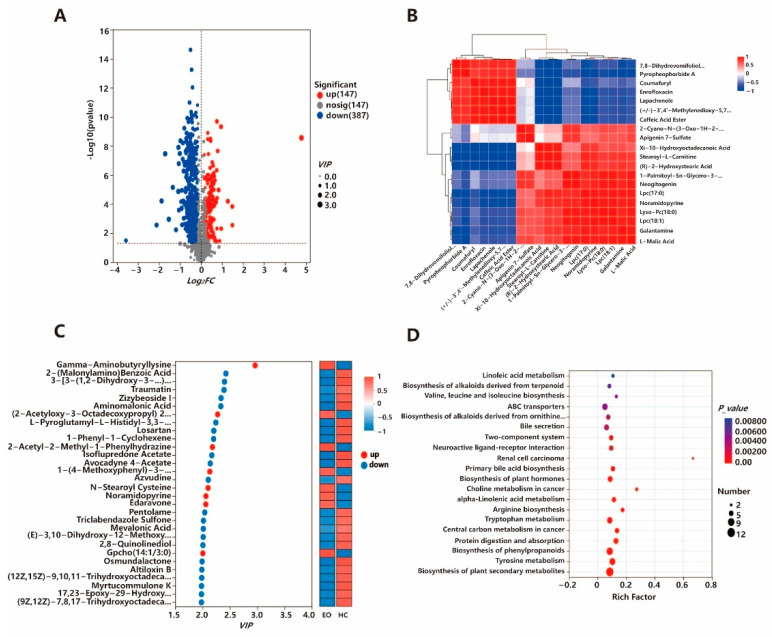
Differential Metabolite Analysis between Eo and HC Groups. (**A**) Volcano plot illustrating the distribution of differential metabolites. (**B**) Heatmap displaying differential metabolites between the Eo and HC groups. (**C**) Bubble chart showing the KEGG enrichment analysis of the differential metabolites. (**D**) Identification of differential metabolites between Eo and HC groups based on VIP scores.

Based on the criteria of *VIP* > 1, *FDR* < 0.05, and |*log*_2_*FC*| > 1, we identified a set of metabolites that exhibited significant differences in expression between the Eo and HC groups (Figure 2A). In total, 543 metabolites showed differential abundance, including 147 that were upregulated and 387 that were downregulated in the Eo group. To explore the interrelationships among these differential metabolites, Pearson correlation analysis was employed. A metabolic network was then constructed by selecting metabolite pairs that met the thresholds of *p*-value < 0.05 and correlation coefficient > 0.95 (Figure 2B). Additionally, we analyzed the top 20 most significant metabolites (ranked by *VIP* score) in the Eo and HC groups (Figure 2C). KEGG pathway enrichment analysis was performed on all differentially expressed metabolites, revealing significant enrichment in pathways such as linoleic acid metabolism; the biosynthesis of alkaloids derived from terpenoid and polyketide, valine, leucine, and isoleucine biosynthesis; and ABC transporters (Figure 2D). Beyond individual pathways, these results indicate that amino acid metabolism (e.g., valine, leucine, and isoleucine biosynthesis), lipid metabolism (e.g., linoleic acid metabolism), and xenobiotic/secondary metabolite pathways (e.g., alkaloid biosynthesis and ABC transporters) were particularly affected, suggesting broad perturbations in nutrient utilization and host–microbe metabolic interactions during coccidial infection. Metabolite abbreviations and their corresponding full names are provided in Table S9.

### 3.6. Integrated Metagenome–Metabolome Analysis

A microbe–metabolite correlation analysis was conducted based on nine representative microbial species identified using LEfSe for the Eo group (*Prevotella* sp., *Fusobacterium necrophorum, Alloprevotella* sp. *OH1205 COT-284*, *Bibersteinia trehalosi*, and *Bacteroides heparinolyticus*) and the HC group (*Ruminococcus* sp., *Clostridium* sp., *Eubacterium* sp., and *Acetatifactor* sp.) together with a set of key metabolites selected for their top 10 *VIP* values.

**Figure 3:**
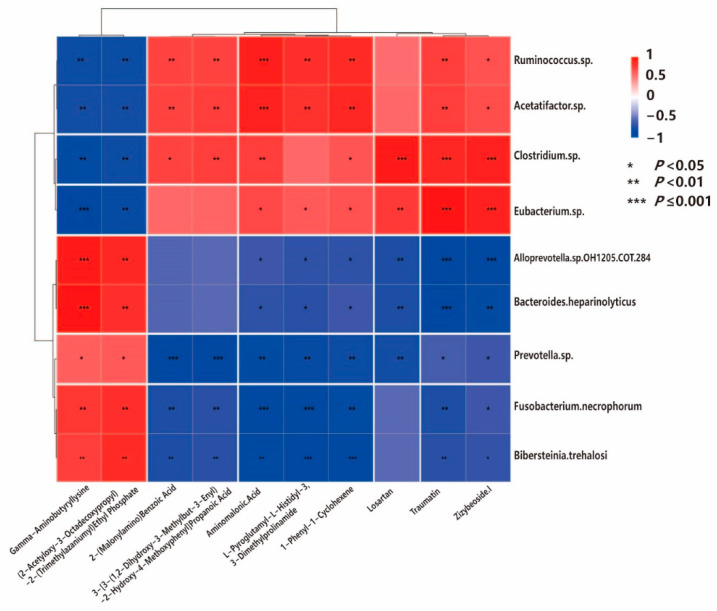
Functional profiles of characteristic microbiomes and metabolomes. Pearson correlation analyses between metabolites and microorganisms are indicated by * (*p* < 0.05), ** (*p* < 0.01), and *** (*p* ≤ 0.001); red squares represent positive correlations, while blue squares represent negative correlations.

In total, 79 statistically significant associations were revealed (*p*_adjusted < 0.05) that demonstrate distinct patterns between Eo-associated and HC-associated bacterial taxa. Among the Eo-enriched bacteria, Bacteroides heparinolyticus showed the strongest positive correlation with Gamma-Aminobutyryllysine (*r* = 0.979, *p*_adjusted = 2.78 × 10^−6^), while *Alloprevotella* sp. OH1205 COT-284 also positively correlated with this metabolite (*r* = 0.937, *p*_adjusted = 1.26 × 10^−4^), suggesting elevated levels of Gamma-Aminobutyryllysine during coccidiosis infection. Conversely, the HC-enriched bacteria *Eubacterium* sp. demonstrated strong negative correlation with Gamma-Aminobutyryllysine (*r* = −0.958, *p*_adjusted = 2.15 × 10^−5^), confirming the metabolite’s association with the diseased state. Traumatin exhibited the opposite pattern, showing strong positive correlations with HC-associated bacteria including *Eubacterium* sp. (*r* = 0.965, *p*_adjusted = 1.16 × 10^−5^), *Clostridium* sp. (*r* = 0.874, *p*_adjusted = 9.51 × 10^−4^), and *Acetatifactor* sp. (*r* = 0.748, *p*_adjusted = 9.04 × 10^−3^), while negatively correlating with Eo-associated *Bacteroides heparinolyticus* (*r* = −0.965, *p*_adjusted = 1.16 × 10^−5^) and *Alloprevotella* sp. (*r* = −0.916, *p*_adjusted = 2.56 × 10^−4^). Similarly, aminomalonic acid showed positive associations with the HC-associated bacteria *Ruminococcus* sp. (*r* = 0.916, *p*_adjusted = 2.56 × 10^−4^) and *Acetatifactor* sp. (*r* = 0.895, *p*_adjusted = 4.71 × 10^−4^), but negative correlations with the Eo-associated *Fusobacterium necrophorum* (*r* = −0.895, *p*_adjusted = 4.71 × 10^−4^) and *Bibersteinia trehalosi* (*r* = −0.860, *p*_adjusted = 1.36 × 10^−3^). Importantly, some metabolites, such as Zizybeoside I and aminomalonic acid, were associated with multiple microbial taxa, suggesting their pivotal roles in the gut microbiome–metabolome network. Overall, microbial species from the Eo group were more likely to be linked to metabolites involved in stress, inflammation, or xenobiotic metabolism, whereas those from the HC group were correlated with metabolites indicative of metabolic stability and homeostasis (Figure 3). The corresponding correlation coefficients (*r* values) are provided in Table S10.

## 4. Discussion

Animals, like humans, function as “superorganisms” due to the symbiotic relationship with their microbial communities [39]. These microbes, especially bacteria, have co-evolved with hosts over millions of years, integrating into key physiological processes from fetal development onward; they influence metabolism, immunity, and neurological functions, playing a vital role in health and adaptation [40,41,42]. Traditional culture-based studies offered limited insights into this complexity; however, advances in high-throughput sequencing now allow comprehensive profiling of the microbiome, revealing its critical roles and associations with diseases such as such as Canine inflammatory bowel disease [43], subacute ruminal acidosis [44], diarrhea and growth disorders in calves [45], and pig post-weaning diarrhea [46].

Metagenomic sequencing technology enables comprehensive, unbiased genome-wide analysis of complex intestinal microbial communities, providing high-resolution insights into microbial population structure, functional pathways, and virulence factor expression. In recent years, metagenomic approaches have been extensively employed to investigate intestinal microecological dysbiosis induced by coccidial infections in poultry and livestock, elucidating the widespread disruption of intestinal microbiota by coccidia and the intricate interactions between microorganisms and their hosts.

### 4.1. Coccidian Infection Is Associated with a Decrease in Intestinal Microbiota Diversity

The sheep gastrointestinal tract serves dual functions in digestion and immune defense [47]. Beyond converting dietary intake into essential nutrients that support growth, development, and reproduction, it acts as a critical barrier against pathogenic colonization through sophisticated pathogen exclusion mechanisms [48].

Our findings revealed that *E. ovinoidalis* infection was associated with a significant reduction in gut microbiota diversity. In Hu lambs experimentally infected with *E. ovinoidalis*, Cheng et al. [7] likewise observed pronounced shifts in community structure and a transient reduction in beneficial taxa and diversity around the peak oocyst shedding period, with partial recovery thereafter, aligning with our observations. This pattern also aligns with previous research by Huang et al. [49], who documented decreased operational taxonomic units (OTUs) in chickens infected with *E. tenella* between 5–7 days post-infection. Their observations of significantly reduced ACE and Shannon indices compared to controls demonstrate that *Eimeria* species infections consistently disrupt host gut microbial communities across different animal species. These convergent findings suggest that coccidial parasites employ similar strategies to alter the intestinal microenvironment, regardless of the specific host–parasite combination.

### 4.2. Coccidiosis-Associated Microbial Imbalance: Depletion of Beneficial Bacteria and Enrichment of Opportunistic Pathogens

The LEfSe analysis revealed substantial differences in the gut microbial communities between the Eo and HC groups. Specifically, members of the genera *Prevotella*, *Fusobacterium*, *Alloprevotella*, *Bacteroides*, and *Bibersteinia* were significantly enriched in the Eo group, while *Ruminococcus*, *Eubacterium*, *Clostridium*, and *Acetatifactor* dominated in the HC group. These shifts may suggest that disease onset is associated with a disruption in microbial homeostasis (dysbiosis) and an increase in potentially pro-inflammatory or opportunistic pathogens. Consistent with our dysbiosis signature of enrichment in opportunistic/pathobiont taxa with concurrent depletion in SCFA producers, recent ovine studies have reported the expansion of *Proteobacteria*/*Actinobacteriota* and reductions in butyrate-associated lineages during *E. ovinoidalis* infection or co-morbid exposures [7,13].

Several taxa enriched in the Eo group have been previously implicated in inflammatory processes. For example, *Fusobacterium necrophorum*, a well-known opportunistic pathogen in ruminants, has been associated with liver abscesses and systemic infections [50]. *Prevotella* species can activate Th17 cells, inducing pro-inflammatory cytokines such as IL-17, which impair intestinal barrier integrity and exacerbate inflammation [51]. Moreover, their mucin-degrading enzymes further compromise the mucosal barrier, facilitating bacterial translocation and immune activation contributing to intestinal inflammation and disease [52,53]. *Bacteroides heparinolyticus*, also known as *Prevotella heparinolytica*, is considered an opportunistic pathogen; it produces heparinase and hyaluronidase enzymes that degrade heparan sulfate in the epithelial basement membrane, increasing epithelial permeability and facilitating bacterial invasion and virulence factor activity [54]. Similarly, *Bibersteinia trehalosi* is commonly isolated from the respiratory tracts of animals with pneumonia and may indicate systemic translocation or a compromised mucosal barrier [55].

In contrast, taxa enriched in the HC group are generally regarded as beneficial microbes involved in gut homeostasis and anti-inflammatory responses. *Ruminococcus* and *Eubacterium* are prominent short-chain fatty acid (SCFA) producers, particularly butyrate, which serves as an energy source for colonocytes and helps reinforce intestinal barrier integrity [56]. Butyrate also possesses immunomodulatory properties, including suppression of pro-inflammatory cytokines and promotion of regulatory T cells (Tregs) via GPR43 and GPR109a signaling pathways [56,57]. Clostridium cluster IV and XIVa members, including certain species detected here, have also been shown to induce colonic Treg differentiation, further supporting immune tolerance and gut health [58,59].

The concurrent depletion of SCFA-producing bacteria and enrichment of potentially pathogenic taxa in the Eo group suggests a pathophysiological mechanism in which dysbiosis may be associated with increased gut permeability, systemic inflammation, and disease progression, creating a vicious cycle of worsening dysbiosis and immune activation. However, these relationships are associative and require further investigation.

### 4.3. Functional Reprogramming of Gut Microbiota: From Homeostatic to Stress-Adapted Communities in Coccidiosis

To explore the functional alterations in the gut microbiota associated with coccidiosis, we performed KEGG-based functional annotation at the gene name level and applied LEfSe to identify significantly enriched microbial functions between the two groups. The Eo group showed marked enrichment in functions related to environmental stress resistance, resource scavenging, and virulence. Notably, susC and susD-outer membrane proteins from the starch utilization system were significantly elevated. While these genes typically enable polysaccharide uptake, they may also be induced under mucosal damage when host-derived glycans become abundant [60]. Similarly, upregulation of tolC and associated efflux proteins (bepC, cyaE, and sapF) points toward increased bacterial tolerance to antimicrobial compounds and host defenses, consistent with a dysbiotic and inflamed gut environment [61].

Furthermore, genes such as hipB (a toxin-antitoxin system component) and dut (dUTPase) are often implicated in bacterial stress survival and DNA damage control [62,63,64]. Both genes are integral to bacterial stress responses, persistence mechanisms, biofilm formation, and managing DNA damage, particularly in pathogenic settings [65,66]. The presence of lemA, a membrane-associated virulence regulator, suggests increased expression of pathogenicity-related elements under infection-induced selective pressure [67].

In contrast, the HC group exhibited enrichment in KEGG gene functions such as yesN (ABC transporter ATP-binding protein), fakB (fatty acid-binding protein), and mutT (NUDIX hydrolase involved in oxidative DNA damage repair). These genes are typically associated with stable, commensal microbial communities and contribute to DNA stability, fatty acid metabolism, and controlled sugar transport [68,69,70]. For example, mutT plays a crucial role in hydrolyzing oxidized nucleotides such as 8-oxo-dGTP, thereby preventing mutagenesis and supporting genomic integrity under mild oxidative conditions [71,72].

Additionally, the HC-enriched gene dacC (D-alanyl-D-alanine carboxypeptidase) participates in peptidoglycan remodeling, contributing to bacterial cell wall maintenance and stability, often linked to slow-growing or homeostatic microbial taxa [73]. Enrichment of regulatory genes like araC also reflects a capacity for adaptive gene expression and efficient utilization of host-derived carbohydrates [74].

Together, these functional patterns suggest that coccidiosis is associated with a shift from a homeostatic, metabolically balanced microbiota toward a more opportunistic, stress-adapted community with enhanced abilities to survive inflammatory conditions, compete for limited resources, and potentially exacerbate disease progression.

### 4.4. Coccidiosis Infection Shifts the Gut Microbiome from Commensal to Competitive Virulence Profiles

To gain insight into how microbial virulence potential differs between Eo and HC animals, we conducted LEfSe analysis based on the Virulence Factor Database (VFDB).

The Eo group exhibited a marked enrichment in bacterial virulence systems, especially secretion-related machinery. Notably, Type IV pili (VF0075) and Type IV secretion systems (VirB/VirD4 VF0369, Trw VF0372) were significantly enriched, supporting the idea that bacteria in infected guts engage in the adhesion, genetic exchange, and direct manipulation of host cells [75]. The presence of Type III secretion systems (TTSS VF0172) and Type VI secretion systems (T6SSs: VF1337, VF0579, and VF0480) further indicates a shift toward competitive and potentially pathogenic behavior. T6SSs, in particular, mediate inter-bacterial antagonism and niche dominance, often correlating with inflammatory disease and microbial dysbiosis [76].

Additionally, the increased expression of Yersiniabactin (VF0136)—a high-affinity iron-chelating siderophore—suggests that bacterial species in the Eo group are competing for trace elements, a hallmark of inflammation-driven environments [77]. The enrichment of tryptophan synthesis (VF0813) may reflect microbial efforts to overcome host-imposed amino acid restriction, given that tryptophan availability is often suppressed during intestinal infection [78].

In contrast, in the HC group, the most discriminative virulence-associated functions included the trehalose-recycling ABC transporter (VF0842), PhoP (VF0286), and multiple capsule synthesis loci (e.g., Capsule VF0274, VF0003, and VF0141). The trehalose system contributes to bacterial stress tolerance, nutrient conservation, and biofilm formation, enabling the long-term survival of commensals in fluctuating environments [79]. Similarly, the PhoP/PhoQ two-component system, while associated with virulence regulation in pathogens, also plays a key role in modulating host interactions and reducing immune recognition under stable gut conditions [80].

The repeated enrichment of capsule-related loci suggests that the microbiota in healthy animals is enriched in bacteria with surface structures that promote mucosal adhesion, biofilm development, and immune evasion—functions that, in the absence of infection, support barrier function and niche protection rather than pathogenicity [81].

Other enriched regulators such as RlrA islet (VF0529) and BvrR-BvrS (VF0368) are typically found in Gram-positive organisms and are linked to adhesion, colonization, and maintenance of host–microbe equilibrium [82]. Additionally, genes involved in amino acid biosynthesis (Glutamine synthesis VF0816) and bile resistance (BSH VF0350) may reflect an active role of the HC microbiota in nutrient regulation and host metabolic balance [83,84,85].

Comparable infection-associated competitive traits—enhanced secretion/adhesion systems and nutrient-acquisition modules—have been described alongside microbiome destabilization in recent *Eimeria* infection models, supporting a shift toward stress-adapted communities under inflammatory pressure [86,87].

These VFDB results indicate that coccidiosis is associated with a shift in the gut microbiome toward a more aggressive, resource-competitive, and inflammation-adapted structure, characterized by the expression of pili, secretion systems, and nutrient acquisition modules. Meanwhile, the healthy microbiota maintains a functionally balanced community expressing traits that promote epithelial tolerance, immune equilibrium, and colonization resistance.

### 4.5. Gut Microbiota–Metabolite Associations Observed in E. ovinoidalis Infection and Stress-Induced Metabolic Imbalance

Several species enriched in the Eo group, including *Prevotella* sp., *Fusobacterium necrophorum*, *Alloprevotella* sp. *OH1205 COT-284*, *Bibersteinia trehalosi*, and Bacteroides heparinolyticus, showed strong correlations with metabolites typically associated with oxidative stress and immune activation. For instance, *Fusobacterium necrophorum* exhibited a strong negative correlation with aminomalonic acid, a non-proteinogenic amino acid known to be elevated under oxidative stress and metabolic dysfunction [88]. This may suggest microbial consumption or a suppressive effect on the accumulation of stress-induced byproducts. Concordantly, ovine studies integrating microbiome–metabolome profiling during coccidial challenge reported coordinated alterations in microbial composition and metabolites, including reduced SCFA-linked pathways and inflammatory-stress metabolites, which substantiate the association patterns we observed [13,89].

*Bacteroides heparinolyticus* was significantly negatively correlated with traumatin, a plant-derived oxylipin with anti-inflammatory and wound-healing properties [90], whereas *Eubacterium* sp. (from the HC group) showed a strong positive correlation. This suggests a microbial shift in the directionality of antioxidant compound modulation in response to infection.

Additionally, *Alloprevotella* sp. showed a negative correlation with Zizybeoside I, a triterpenoid compound of botanical origin with known anti-inflammatory effects [91], potentially reflecting altered biotransformation pathways under coccidial-induced inflammation.

Microbial taxa from the HC group, including *Ruminococcus* sp., *Clostridium* sp., *Eubacterium* sp., and *Acetatifactor* sp., displayed correlations consistent with a more balanced and homeostatic metabolic profile. Notably, *Eubacterium* sp. was positively correlated with traumatin and negatively with gamma-aminobutyryllysine, a GABA-like metabolite that plays a role in gut–brain signaling [92]. The balance of these molecules may reflect microbial regulation of anti-inflammatory and neuromodulatory pathways.

*Acetatifactor* sp. showed strong positive correlations with 1-phenyl-1-cyclohexene and Zizybeoside I, which may indicate a role in modulating plant-derived or lipid-related bioactive compounds involved in barrier integrity and host immunity [93]. These microbes are widely recognized for their contributions to short-chain fatty acid (SCFA) production, the regulation of mucosal immunity, and the maintenance of intestinal barrier function [94].

Several metabolites, such as gamma-aminobutyryllysine, Zizybeoside I, and aminomalonic acid, were correlated in opposite directions depending on the microbial origin group. For instance, Gamma-aminobutyryllysine showed a strong positive correlation with *Bacteroides heparinolyticus* (Eo group) and a negative correlation with *Clostridium* sp. and *Acetatifactor* sp. (HC group). This inverse pattern suggests competing microbial regulation of host signaling molecules involved in stress and inflammation. Likewise, Zizybeoside I was positively associated with most microbes in the HC group but negatively with those in the infected group, implying an altered metabolic capacity or impaired anti-inflammatory buffering during infection. This shift could represent a microbial functional switch from collaborative homeostasis to antagonistic dysregulation under pathogenic pressure.

This work has several limitations. First, the sample size was very small (*n* = 2 per group). Although biological triplicates were included during sequencing to enhance reliability, the limited biological replication inevitably reduces statistical power and restricts the generalizability of our findings. Second, although the use of non-parametric statistical methods (Wilcoxon test) was appropriate, the risk of Type II errors remains, and potential overfitting may occur in multivariate analyses such as OPLS-DA and correlation analyses. Third, no bacterial strains were isolated in this study, which limited our ability to validate the observed associations and elucidate the underlying biological mechanisms in animal models. Taken together, these limitations highlight the need for future studies with larger sample sizes, more diverse populations, and experimental validation in animal models to confirm causality and further clarify the mechanistic links between altered gut microbiota, metabolite profiles, and disease progression.

## 5. Conclusions

Coccidiosis infection comprehensively disrupted the intestinal ecosystem through multiple interconnected mechanisms. This involved significantly reducing microbiota diversity, inducing microbial imbalance characterized by beneficial bacteria depletion and opportunistic pathogen enrichment, functionally reprogramming gut communities from homeostatic to stress-adapted profiles, shifting microbiome composition toward competitive virulence phenotypes, and establishing strong gut microbiota–metabolite associations that drive *E. ovinoidalis*-mediated metabolic imbalance. These findings demonstrate that coccidiosis creates a cascade of pathophysiological disruption extending from initial parasitic infection through microbial dysbiosis to systemic metabolic dysfunction, providing compelling evidence that altered microbiota and metabolic signatures represent key disease mechanisms and potential biomarkers for improved coccidiosis diagnosis and therapeutic intervention in livestock production systems.

## Data Availability

The datasets supporting the conclusions of this article are included within the article and its supplementary files. Additional data that support the findings of this study are available from the corresponding author upon reasonable request.

## Supplementary Materials

The following supporting information can be downloaded at: https://www.mdpi.com/article/10.3390/biology14091190/s1, Table S1: Primer sequences used for detecting pathogens; Table S2: Summary of raw metagenomic sequencing reads per sample; Table S3: Quality-controlled and host-filtered reads retained per sample; Table S4: De novo assembled contigs statistics per sample; Table S5: Taxonomic composition of the metagenomic dataset; Table S6: LEfSe-identified differential bacteria taxa between Eo and HC groups; Table S7: LEfSe-identified differential KEGG functions between Eo and HC groups; Table S8: LEfSe-identified differential virulence factors between Eo and HC groups; Table S9: Abbreviations and full names of metabolites used in this study; Table S10: Correlation analysis between microbes and metabolites.

## References

[1] de Macedo L.O., Bezerra-Santos M.A., de Mendonça C.L., Alves L.C., Ramos R.A.N., de Carvalho G.A. (2020). Prevalence and risk factors associated with infection by Eimeria spp. in goats and sheep in Northeastern Brazil. J. Parasit. Dis..

[2] Mohammed N.H., Alobaidii W.A., Hasan M.H. (2021). Coccidiosis In Sheep And Goats (Review). Assiut Vet. Med. J..

[3] Chartier C., Paraud C. (2012). Coccidiosis due to Eimeria in sheep and goats, a review. Small Rumin. Res..

[4] Gül A., Değer S. (2002). The Prevalance and Distribution of Eimeria Species Found in Sheep in Van. Turk. J. Vet. Anim. Sci..

[5] Mohamaden W.I., Sallam N.H., Abouelhassan E.M. (2018). Prevalence of Eimeria species among sheep and goats in Suez Governorate, Egypt. Int. J. Vet. Sci. Med..

[6] Olmos L., Caro L.C., Avellaneda-Cáceres A., Medina D., Sandoval V., Aguirre D., Micheloud J. (2020). First record of clinical coccidiosis (Eimeria ovinoidalis) in adult sheep from northwestern Argentina. Vet. Parasitol. Reg. Stud. Rep..

[7] Cheng S., Wang N., Wang C., Liu S., Li S., Li D., Zhang S., Xu H., Zhang L., Jian F. (2024). Impacts of a highly pathogenic ovine Eimeria ovinoidalis on the growth of Hu lambs. Vet. Parasitol..

[8] Einarsson E., Ma’ayeh S., Svärd S.G. (2016). An up-date on Giardia and giardiasis. Current Opinion in Microbiology.

[9] Wang J., Zhu N., Su X., Gao Y., Yang R. (2023). Gut-Microbiota-Derived Metabolites Maintain Gut and Systemic Immune Homeostasis. Cells.

[10] Wiertsema S.P., van Bergenhenegouwen J., Garssen J., Knippels L.M.J. (2021). The Interplay between the Gut Microbiome and the Immune System in the Context of Infectious Diseases throughout Life and the Role of Nutrition in Optimizing Treatment Strategies. Nutrients.

[11] Ding G., Yang X., Li Y., Wang Y., Du Y., Wang M., Ye R., Wang J., Zhang Y., Chen Y. (2024). Gut microbiota regulates gut homeostasis, mucosal immunity and influences immune-related diseases. Mol. Cell. Biochem..

[12] Zhang R., Ding N., Feng X., Liao W. (2025). The gut microbiome, immune modulation, and cognitive decline: Insights on the gut-brain axis. Front. Immunol..

[13] Huang S.-C., Liu K.-L., Chen P., Xu B.-W., Ding W.-L., Yue T.-J., Lu Y.-N., Li S.-Y., Li J.-K., Jian F.-C. (2024). New insights into the combined effects of aflatoxin B1 and Eimeria ovinoidalis on uterine function by disrupting the gut-blood-reproductive axis in sheep. Microbiome.

[14] Wang Q., Wang K., Wu W., Giannoulatou E., Ho J.W., Li L. (2019). Host and microbiome multi-omics integration: Applications and methodologies. Biophys. Rev..

[15] Wu J., Singleton S.S., Bhuiyan U., Krammer L., Mazumder R. (2024). Multi-omics approaches to studying gastrointestinal microbiome in the context of precision medicine and machine learning. Front. Mol. Biosci..

[16] Chetty A., Blekhman R. (2024). Multi-omic approaches for host-microbiome data integration. Gut Microbes.

[17] Duan D., Wang M., Han J., Li M., Wang Z., Zhou S., Xin W., Li X. (2025). Advances in multi-omics integrated analysis methods based on the gut microbiome and their applications. Front. Microbiol..

[18] Schirmer M., Stražar M., Avila-Pacheco J., Rojas-Tapias D.F., Brown E.M., Temple E., Deik A., Bullock K., Jeanfavre S., Pierce K. (2024). Linking microbial genes to plasma and stool metabolites uncovers host-microbial interactions underlying ulcerative colitis disease course. Cell Host Microbe.

[19] Li R.W., Liu F., Solano-Aguilar G., Urban J.F. (2024). 40 The gut microbiota modifies host-parasite interactions. J. Anim. Sci..

[20] Su F., Su M., Wei W., Wu J., Chen L., Sun X., Liu M., Sun S., Mao R., Bourgonje A.R. (2025). Integrating multi-omics data to reveal the host-microbiota interactome in inflammatory bowel disease. Gut Microbes.

[21] Sloss M.W., Kemp R.L. (1994). Veterinary Clinical Parasitology.

[22] Xiao L.H., Morgan U.M., Limor J., Escalante A., Lal A.A. (1999). Genetic Diversity within Cryptosporidium parvum and Related Cryptosporidium Species. Appl. Environ. Microbiol..

[23] Appelbee A.J., Frederick L.M., Heitman T.L., Olson M.E. (2003). Prevalence and genotyping of Giardia duodenalis from beef calves in Alberta, Canada. Vet. Parasitol..

[24] Soares V.M., dos Santos E.A.R., Tadielo L.E., Cerqueira-Cézar C.K., da Cruz Encide Sampaio A.N., Eisen A.K.A., de Oliveira K.G., Padilha M.B., de Moraes Guerra M.E., Gasparetto R. (2022). Detection of adenovirus, rotavirus, and hepatitis E virus in meat cuts marketed in Uruguaiana, Rio Grande do Sul, Brazil. One Health.

[25] Ursula C.O., Jane S.F., Dominique L., Michal P., Arthur G. (2010). Development of molecular assays for the identification of the 11 Eimeria species of the domestic rabbit (*Oryctolagus cuniculus*). Vet. Parasitol..

[26] Gomes-dos-Santos A., Fonseca E., Riccardi N., Hinzmann M., Lopes-Lima M., Froufe E. (2024). The transcriptome assembly of the European freshwater mussel Unio elongatulus C. Pfeiffer, 1825. Sci. Data.

[27] Bolger A.M., Lohse M., Usadel B. (2014). Trimmomatic: A flexible trimmer for Illumina sequence data. Bioinformatics.

[28] Langmead B., Salzberg S.L. (2012). Fast gapped-read alignment with Bowtie 2. Nat. Methods.

[29] Li D., Liu C.-M., Luo R., Sadakane K., Lam T.-W. (2015). MEGAHIT: An ultra-fast single-node solution for large and complex metagenomics assembly via succinct de Bruijn graph. Bioinformatics.

[30] Hyatt D., Chen G.-L., LoCascio P.F., Land M.L., Larimer F.W., Hauser L.J. (2010). Prodigal: Prokaryotic gene recognition and translation initiation site identification. BMC Bioinform..

[31] Fu L., Niu B., Zhu Z., Wu S., Li W. (2012). CD-HIT: Accelerated for clustering the next-generation sequencing data. Bioinformatics.

[32] Huson D.H., Buchfink B. (2015). Fast and sensitive protein alignment using DIAMOND. Nat. Methods.

[33] Coordinators N.R. (2015). Database resources of the National Center for Biotechnology Information. Nucleic Acids Res..

[34] Segata N., Izard J., Waldron L., Gevers D., Miropolsky L., Garrett W.S., Huttenhower C. (2011). Metagenomic biomarker discovery and explanation. Genome Biol..

[35] Minoru K., Susumu G., Yoko S., Masayuki K., Miho F., Mao T. (2013). Data, information, knowledge and principle: Back to metabolism in KEGG. Nucleic Acids Res..

[36] Chen L., Yang J., Yu J., Yao Z., Sun L., Shen Y., Jin Q. (2004). VFDB: A reference database for bacterial virulence factors. Nucleic Acids Res..

[37] Liu F., Smith A.D., Solano-Aguilar G., Wang T.T.Y., Li R.W. (2020). Mechanistic insights into the attenuation of intestinal inflammation and modulation of the gut microbiome by krill oil using in vitro and in vivo models. Microbiome.

[38] Smith C.A., Want E.J., O’Maille G., Ruben Abagyan A., Siuzdak G. (2006). XCMS: Processing Mass Spectrometry Data for Metabolite Profiling Using Nonlinear Peak Alignment, Matching, and Identification. Anal. Chem..

[39] Zhu B., Wang X., Li L. (2010). Human gut microbiome: The second genome of human body. Protein Cell.

[40] Alegado R.A., King N. (2014). Bacterial Influences on Animal Origins. Cold Spring Harb. Perspect. Biol..

[41] Kostic A.D., Howitt M.R., Garrett W.S. (2013). Exploring host–microbiota interactions in animal models and humans. Genes Dev..

[42] McFall-Ngai M., Hadfield M.G., Bosch T.C.G., Carey H.V., Domazet-Lošo T., Douglas A.E., Dubilier N., Eberl G., Fukami T., Gilbert S.F. (2013). Animals in a bacterial world, a new imperative for the life sciences. Proc. Natl. Acad. Sci. USA.

[43] Minamoto Y., Otoni C.C., Steelman S.M., Büyükleblebici O., Steiner J.M., Jergens A.E., Suchodolski J.S. (2014). Alteration of the fecal microbiota and serum metabolite profiles in dogs with idiopathic inflammatory bowel disease. Gut Microbes.

[44] Plaizier J.C., Krause D.O., Gozho G.N., McBride B.W. (2008). Subacute ruminal acidosis in dairy cows: The physiological causes, incidence and consequences. Vet. J..

[45] Malmuthuge N., Griebel P.J., Guan L.L. (2015). The Gut Microbiome and Its Potential Role in the Development and Function of Newborn Calf Gastrointestinal Tract. Front. Vet. Sci..

[46] Gresse R., Chaucheyras-Durand F., Fleury M.A., Van de Wiele T., Forano E., Blanquet-Diot S. (2017). Gut Microbiota Dysbiosis in Postweaning Piglets: Understanding the Keys to Health. Trends Microbiol..

[47] Lu C., Yan Y., Jian F., Ning C. (2021). Coccidia-Microbiota Interactions and Their Effects on the Host. Front. Cell. Infect. Microbiol..

[48] Thabile M., Moses O., Matthew Adekunle A. (2021). Understanding the interactions between Eimeria infection and gut microbiota, towards the control of chicken coccidiosis: A review. Parasite.

[49] Huang G., Tang X., Bi F., Hao Z., Han Z., Suo J., Zhang S., Wang S., Duan C., Yu Z. (2018). Eimeria tenella infection perturbs the chicken gut microbiota from the onset of oocyst shedding. Vet. Parasitol..

[50] Amachawadi R.G., Tom W.A., Hays M.P., Fernando S.C., Hardwidge P.R., Nagaraja T.G. (2021). Bacterial community analysis of purulent material from liver abscesses of crossbred cattle and Holstein steers fed finishing diets with or without tylosin. J. Anim. Sci..

[51] Huang Y., Tang J., Cai Z., Zhou K., Chang L., Bai Y., Ma Y. (2020). Prevotella Induces the Production of Th17 Cells in the Colon of Mice. J. Immunol. Res..

[52] Glover J.S., Ticer T.D., Engevik M.A. (2022). Characterizing the mucin-degrading capacity of the human gut microbiota. Sci. Rep..

[53] Wright D.P., Rosendale D.I., Roberton A.M. (2000). Prevotella enzymes involved in mucin oligosaccharide degradation and evidence for a small operon of genes expressed during growth on mucin. FEMS Microbiol. Lett..

[54] Sun J., Xu X., Gao S., Pan Q., Liu Z., Huang Y., Lian Y. (2024). Refractory pneumonia caused by Prevotella heparinolytica: A case report. J. Med. Case Rep..

[55] Wood M.E., Fox K.A., Jennings-Gaines J., Killion H.J., Amundson S., Miller M.W., Edwards W.H. (2017). How Respiratory Pathogens Contribute to Lamb Mortality in a Poorly Performing Bighorn Sheep (Ovis canadensis) Herd. J. Wildl. Dis..

[56] Singh V., Lee G., Son H., Koh H., Kim E.S., Unno T., Shin J.-H. (2023). Butyrate producers,”The Sentinel of Gut”: Their intestinal significance with and beyond butyrate, and prospective use as microbial therapeutics. Front. Microbiol..

[57] Zhang D., Jian Y.-P., Zhang Y.-N., Li Y., Gu L.-T., Sun H.-H., Liu M.-D., Zhou H.-L., Wang Y.-S., Xu Z.-X. (2023). Short-chain fatty acids in diseases. Cell Commun. Signal..

[58] Narushima S., Sugiura Y., Oshima K., Atarashi K., Hattori M., Suematsu M., Honda K. (2014). Characterization of the 17 strains of regulatory T cell-inducing human-derived Clostridia. Gut Microbes.

[59] Atarashi K., Tanoue T., Shima T., Imaoka A., Kuwahara T., Momose Y., Cheng G., Yamasaki S., Saito T., Ohba Y. (2011). Induction of colonic regulatory T cells by indigenous Clostridium species. Science.

[60] Martens E.C., Koropatkin N.M., Smith T.J., Gordon J.I. (2009). Complex glycan catabolism by the human gut microbiota: The Bacteroidetes Sus-like paradigm. J. Biol. Chem..

[61] Piddock L.J. (2006). Multidrug-resistance efflux pumps—not just for resistance. Nat. Rev. Microbiol..

[62] Singh G., Yadav M., Ghosh C., Rathore J.S. (2021). Bacterial toxin-antitoxin modules: Classification, functions, and association with persistence. Curr. Res. Microb. Sci..

[63] Wen Y., Behiels E., Devreese B. (2014). Toxin-Antitoxin systems: Their role in persistence, biofilm formation, and pathogenicity. Pathog. Dis..

[64] Dawan J., Ahn J. (2022). Bacterial Stress Responses as Potential Targets in Overcoming Antibiotic Resistance. Microorganisms.

[65] Sonika S., Singh S., Mishra S., Verma S. (2023). Toxin-antitoxin systems in bacterial pathogenesis. Heliyon.

[66] Karimi S., Ghafourian S., Taheri Kalani M., Azizi Jalilian F., Hemati S., Sadeghifard N. (2014). Association Between Toxin-Antitoxin Systems and Biofilm Formation. Jundishapur J. Microbiol..

[67] Ostyn E., Augagneur Y., Pinel-Marie M.L. (2025). Insight into the environmental cues modulating the expression of bacterial toxin-antitoxin systems. FEMS Microbiol. Rev..

[68] Qu Q., Peng H., Chen M., Liu X., Che R., Bello-Onaghise G., Zhang Z., Chen X., Li Y. (2024). The relationship between resistance evolution and carbon metabolism in Staphylococcus xylosus under ceftiofur sodium stress. Arch. Microbiol..

[69] Poetsch A.R. (2020). The genomics of oxidative DNA damage, repair, and resulting mutagenesis. Comput. Struct. Biotechnol. J..

[70] Xiao Q., Xia M., Tang W., Zhao H., Chen Y., Zhong J. (2024). The lipid metabolism remodeling: A hurdle in breast cancer therapy. Cancer Lett..

[71] Arczewska K.D., Kuśmierek J.T. (2007). Bacterial DNA repair genes and their eukaryotic homologues: 2. Role of bacterial mutator gene homologues in human disease. Overview of nucleotide pool sanitization and mismatch repair systems. Acta Biochim. Pol..

[72] Lu A.L., Li X., Gu Y., Wright P.M., Chang D.-Y. (2001). Repair of Oxidative DNA Damage: Mechanisms and Functions. Cell Biochem. Biophys..

[73] Typas A., Banzhaf M., Gross C.A., Vollmer W. (2012). From the regulation of peptidoglycan synthesis to bacterial growth and morphology. Nat. Rev. Microbiol..

[74] Robert S. (2010). AraC protein, regulation of the l-arabinose operon in Escherichia coli, and the light switch mechanism of AraC action. FEMS Microbiol. Rev..

[75] Christie P.J., Cascales E. (2003). The versatile bacterial type IV secretion systems. Nat. Rev. Microbiol..

[76] Russell A.B., Peterson S.B., Mougous J.D. (2014). Type VI secretion system effectors: Poisons with a purpose. Nat. Rev. Microbiol..

[77] Bachman M.A., Oyler J.E., Burns S.H., Caza M., Lépine F., Dozois C.M., Weiser J.N., Bäumler A.J. (2011). Klebsiella pneumoniae yersiniabactin promotes respiratory tract infection through evasion of lipocalin 2. Infect. Immun..

[78] Zelante T., Iannitti R.G., Cunha C., De Luca A., Giovannini G., Pieraccini G., Zecchi R., D’Angelo C., Massi-Benedetti C., Fallarino F. (2013). Tryptophan catabolites from microbiota engage aryl hydrocarbon receptor and balance mucosal reactivity via interleukin-22. Immunity.

[79] Avonce N., Mendoza-Vargas A., Morett E., Iturriaga G. (2006). Insights on the evolution of trehalose biosynthesis. BMC Evol. Biol..

[80] Groisman E.A. (2001). The Pleiotropic Two-Component Regulatory System PhoP-PhoQ. J. Bacteriol..

[81] Rendueles O., Ghigo J.M. (2015). Mechanisms of Competition in Biofilm Communities. Microbiol. Spectr..

[82] Tettelin H., Masignani V., Cieslewicz M., Donati C., Medini D., Ward N., Angiuoli S., Crabtree J., Jones A., Durkin A. (2005). Genome analysis of multiple pathogenic isolates of Streptococcus agalactiae: Implications for the microbial “pan-genome”. Proc. Natl. Acad. Sci. USA.

[83] Joyce S.A., Shanahan F., Hill C., Gahan C.G.M. (2014). Bacterial bile salt hydrolase in host metabolism: Potential for influencing gastrointestinal microbe-host crosstalk. Gut Microbes.

[84] Agolino G., Cristofolini M., Vaccalluzzo A., Tagliazucchi D., Cattivelli A., Pino A., Caggia C., Solieri L., Randazzo C.L. (2025). Genome Mining and Characterization of Two Novel Lacticaseibacillus rhamnosus Probiotic Candidates with Bile Salt Hydrolase Activity. Biomolecules.

[85] Chwals W. (2004). Regulation of the Cellular and Physiological Effects of Glutamine. Mini-Rev. Med. Chem..

[86] Liu J., Guo J., Whitmore Melanie A., Tobin I., Kim Dohyung M., Zhao Z., Zhang G. (2024). Dynamic response of the intestinal microbiome to Eimeria maxima-induced coccidiosis in chickens. Microbiol. Spectr..

[87] Tang J., Wang Q., Yu H., Dong L., Tang M., Arif A., Zhang G., Zhang T., Xie K., Su S. (2024). A Comparison of the Cecal Microbiota between the Infection and Recovery Periods in Chickens with Different Susceptibilities to Eimeria tenella. Animals.

[88] Taranto D., Kloosterman D.J., Akkari L. (2024). Macrophages and T cells in metabolic disorder-associated cancers. Nat. Rev. Cancer.

[89] Liu S., Li S., Cheng S., Liu M., Li J., Li S., Li X., Zhang L., Jian F. (2025). Effect of Artemisia annua on anticoccidial action, intestinal microbiota and metabolites of Hu lambs. BMC Vet. Res..

[90] Kallenbach M., Gilardoni P.A., Allmann S., Baldwin I.T., Bonaventure G. (2011). C_12_ derivatives of the hydroperoxide lyase pathway are produced by product recycling through lipoxygenase-2 in *Nicotiana attenuata* leaves. New Phytol..

[91] Ravanan P., Singh S.K., Rao G.S.R.S., Kondaiah P. (2011). Growth inhibitory, apoptotic and anti-inflammatory activities displayed by a novel modified triterpenoid, cyano enone of methyl boswellates. J. Biosci..

[92] Braga J.D., Thongngam M., Kumrungsee T. (2024). Gamma-aminobutyric acid as a potential postbiotic mediator in the gut–brain axis. npj Sci. Food.

[93] Nicholson J.K., Holmes E., Wilson I.D. (2005). Gut microorganisms, mammalian metabolism and personalized health care. Nat. Rev. Microbiol..

[94] Rowland I., Gibson G., Heinken A., Scott K., Swann J., Thiele I., Tuohy K. (2018). Gut microbiota functions: Metabolism of nutrients and other food components. Eur. J. Nutr..

