# Supplementary material for: Eimeria ovinoidalis Infection Reshapes Gut Microbial Communities and Metabolic Profiles in Tan Sheep
Source: Biology (Basel). 2025 Sep 4;14(9):1190. doi: 10.3390/biology14091190 (PMC12467318; doi:10.3390/biology14091190)
Supplement: Supplementary file 1 [file biology-14-01190-s001.zip › biology-3816335-supplementary.pdf]

Table S1 Primer sequences used for detecting pathogens

Target Pathogen	Primer sequences (5′-3′)	Fragment size (bp)	Temperature (°C)	References
Cryptosporidium	18sU1:TTCTAGAGCTAATACATGCG<br>18SD1:CCCATTTCCTTCGAAACAGGA	1300	58	Xiao et al.[1]
	18SU2:GGAAGGGTTGTATTTATTAGATAAAG<br>18SD2:AAGGAGTAAGGAACAACCTCCA	830	55	
Giardia duodenalis	Gia2029:AAGTGTGGTGCAGACGGACTC<br>Gia2150c:CTGCTGCCGTCCTTGGATGT		55	Appelbee et al.[2]
	RH11:CATCCGGTCGATCCTGCC<br>RH4：AGTCGAACCCTGATTCTCCGCCCAGG	292	59	
Rotavirus	RVF:GATGTCCTGTACTCCTTGT<br>RVR:GGTAGATTACCAATTCCTCC	160	50	Soares VM et al.[3]
Eimeria spp.	ITS-1F：GGGAAGTTGCGTAAATAGA<br>ITS-1R: CTGCGTCCTTCATCGAT	~640	53	Oliveira U.C. et al.[4]

References

1. Xiao LH, Morgan UM, Limor J, Escalante A, Lal AA: Genetic Diversity within Cryptosporidium parvum and Related Cryptosporidium Species. Applied and Environmental Microbiology 1999, 65(8):3386-3391.
2. A J A, L M F, T L H, M E O: Prevalence and genotyping of Giardia duodenalis from beef calves in Alberta, Canada. Vet Parasitol 2003, 112(4).
3. Vanessa Mendonça S, Emanoelli Aparecida Rodrigues DS, Leonardo Ereno T, Camila Koutsodontis C-C, Aryele Nunes dCES, Ana Karolina Antunes E, Kelen Gras dO, Matheus Beltrame P, Maria Eduarda dMG, Raíssa G et al: Detection of adenovirus, rotavirus, and hepatitis E virus in meat cuts marketed in Uruguaiana, Rio Grande do Sul, Brazil. One Health 2022, 14(0).
4. Ursula C O, Jane S F, Dominique L, Michal P, Arthur G: Development of molecular assays for the identification of the 11 Eimeria species of the domestic rabbit (Oryctolagus cuniculus). Vet Parasitol 2010, 176(0).
